# Crystal structure and Hirshfeld surface analysis of 2-amino-4-(4-meth­oxy­phen­yl)-6-oxo-1-phenyl-1,4,5,6-tetra­hydro­pyridine-3-carbo­nitrile

**DOI:** 10.1107/S205698902200175X

**Published:** 2022-02-22

**Authors:** Khammed A. Asadov, Victor N. Khrustalev, Ekaterina V. Dobrokhotova, Mehmet Akkurt, Afet T. Huseynova, Anzurat A. Akobirshoeva, Elnur Z. Huseynov

**Affiliations:** a Department of Chemistry, Baku State University, Z. Khalilov str. 23, Az, 1148 Baku, Azerbaijan; b Peoples’ Friendship University of Russia (RUDN University), Miklukho-Maklay St. 6, Moscow, 117198, Russian Federation; c N. D. Zelinsky Institute of Organic Chemistry RAS, Leninsky Prosp. 47, Moscow, 119991, Russian Federation; d Department of Physics, Faculty of Sciences, Erciyes University, 38039 Kayseri, Turkey; e Acad Sci Republ Tadzhikistan, Kh. Yu. Yusufbekov Pamir Biol. Inst., 1 Kholdorova St, Khorog 736002, Gbao, Tajikistan

**Keywords:** crystal structure, tetra­hydro­pyridine, hydrogen bonds, dimers, Hirshfeld surface analysis

## Abstract

The central tetra­hydro­pyridine ring of the title compound, C_19_H_17_N_3_O_2_, adopts a screw-boat conformation. In the crystal, strong C—H⋯O and N—H⋯N hydrogen bonds form dimers with *R* ^2^_2_(14) and *R* ^2^_2_(12) ring motifs, respectively, between consecutive mol­ecules along the *c*-axis direction. Inter­molecular N—H⋯O and C—H⋯O hydrogen bonds connect these dimers, forming a three-dimensional network. C—H⋯π inter­actions and π–π stacking inter­actions contribute to the stabilization of the mol­ecular packing. A Hirshfeld surface analysis indicates that the contributions from the most prevalent inter­actions are H⋯H (47.1%), C⋯H/H⋯C (20.9%), O⋯H/H⋯O (15.3%) and N⋯H/H⋯N (11.4%).

## Abstract 2

In the crystal, strong C—H⋯O and N—H⋯N hydrogen bonds form dimers with 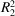 (14) and 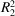 (12) ring motifs between adjacent mol­ecules along the *c*-axis direction. Inter­molecular N—H⋯O and C—H⋯O hydrogen bonds connect these dimers to form a three-dimensional network. In addition, C—H⋯π inter­actions and π–π stacking inter­actions help to stabilize the packing.

## Chemical context

Carbon–carbon and carbon–nitro­gen bond-forming reactions represent an important synthetic class in organic chemistry (Yadigarov *et al.*, 2009 ▸; Abdelhamid *et al.*, 2011 ▸; Yin *et al.*, 2020 ▸; Khalilov *et al.*, 2021 ▸). Notably, pyridine derivatives are widely applied in the discovery of biologically active mol­ecules and multifunctional materials (Magerramov *et al.*, 2018 ▸; Sherman & Murugan, 2015 ▸; Mamedov *et al.*, 2020 ▸). On the other hand, the tetra­hydro­pyridine moiety is an essential part of diverse biologically active compounds, food additives and natural products (Mateeva *et al.*, 2005 ▸). 
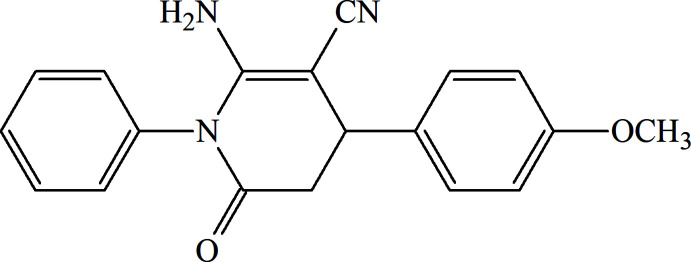


In the framework of ongoing structural studies (Safavora *et al.*, 2019 ▸; Naghiyev *et al.*, 2020 ▸; 2021*a* ▸,*b* ▸; Maharramov *et al.*, 2021 ▸), we report here the crystal structure and Hirshfeld surface analysis of the title compound, 2-amino-4-(4-meth­oxy­phen­yl)-6-oxo-1-phenyl-1,4,5,6-tetra­hydro­pyridine-3- carbo­nitrile.

## Structural commentary

**Figure 1:**
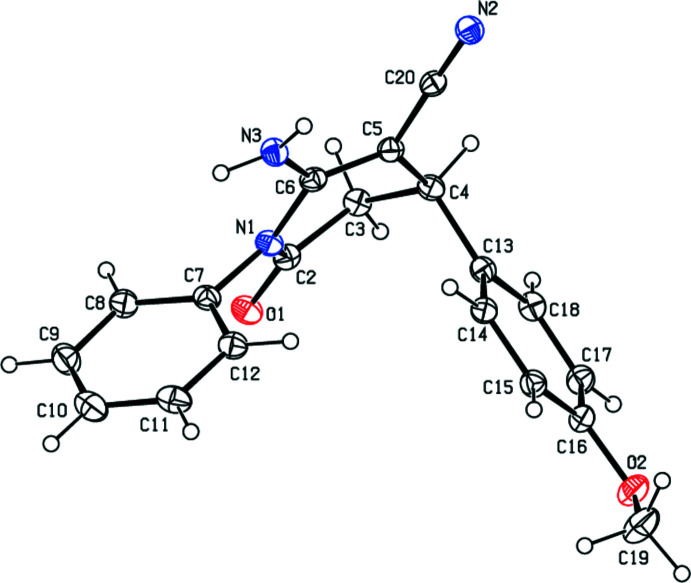
The mol­ecular structure of the title compound with displacement ellipsoids drawn at the 30% probability level.

The title compound (Fig. 1 ▸) crystallizes in the monoclinic space group *P*2_1_/*n* with *Z* = 4. The central N1/C2–C6 tetra­hydro­pyridine ring of the mol­ecule adopts a screw-boat conformation with puckering parameters (Cremer & Pople, 1975 ▸) *Q*_T_ = 0.503 (2) Å, θ = 66.1 (2)°, φ = 153.3 (2)°. The C7–C12 phenyl ring, which is attached to N1, is in an equatorial position and makes a dihedral angle of 54.43 (9)° with the mean plane of the tetra­hydro­pyridine ring. The C13–C18 meth­oxy­phenyl ring, which is attached to C4, is in an axial position. The dihedral angle between the C7–C12 phenyl and C13–C18 meth­oxy­phenyl rings is 68.61 (10)°.

## Supra­molecular features

**Figure 2:**
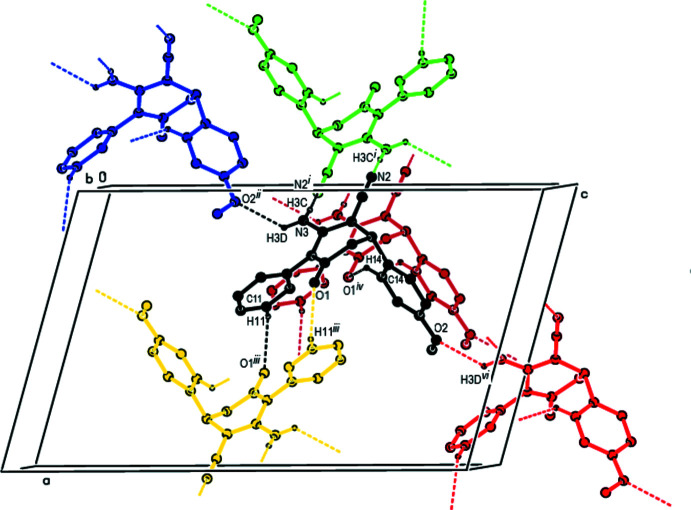
A general view of the N—H⋯N, N—H⋯O and C—H⋯O hydrogen bonds in the crystal packing of the title compound [symmetry codes: (i) −*x*, −*y*, −*z* + 1; (ii) *x* − 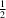 , −*y* + 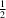 , *z* − 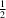 ; (iii) −*x* + 1, −*y* + 1, −*z* + 1; (iv) *x*, *y* − 1, *z*; (vi) 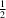 + *x*, 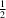 − *y*, 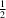 + *z*].

**Figure 3:**
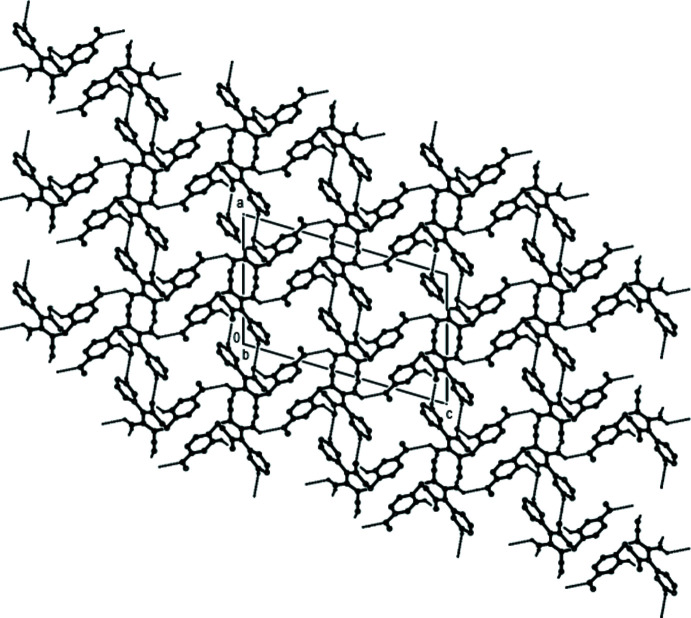
The crystal packing of the title compound, viewed along the *b* axis, showing the N—H⋯N, N—H⋯O and C—H⋯O hydrogen bonds as dashed lines.

**Figure 4:**
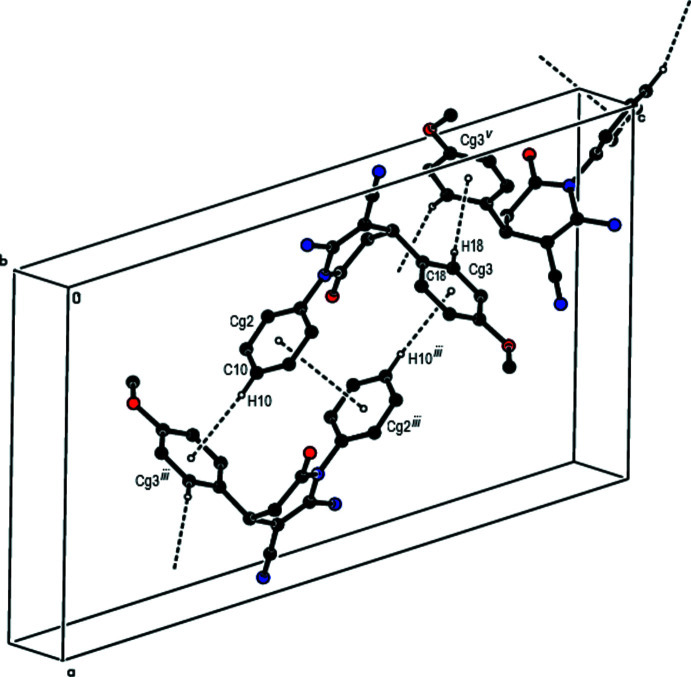
A general view of the C—H⋯π inter­actions and π–π stacking inter­actions in the crystal packing of the title compound [symmetry codes: (iii) −*x* + 1, −*y* + 1, −*z* + 1; (v) −*x* + 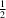 , *y* + 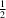 , −*z* + 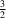 ].

As shown in Fig. 2 ▸, strong inter­molecular C11—H11⋯O1 and N3—H3*C*⋯N2 hydrogen bonds (Table 1 ▸) form dimers with 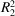 (14) and 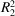 (12) ring motifs (Bernstein *et al.*, 1995 ▸), respectively, between adjacent mol­ecules along the *c*-axis direction. These dimers are connected by N3—H3*D*⋯O2 and C14—H14⋯O1 hydrogen bonds, forming a three-dimensional network (Table 1 ▸; Fig. 3 ▸). Furthermore, C—H⋯π [C10—H10⋯*Cg*3^iii^ and C18—H18⋯*Cg*3^v^; symmetry codes: (iii) −*x* + 1, −*y* + 1, −*z* + 1; (v) −*x* + 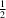 , *y* + 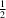 , −*z* + 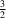 .; *Cg*3 is the centroid of the C13–C18 meth­oxy­phenyl ring; Table 1 ▸] and π–π stacking inter­actions [*Cg*2⋯*Cg*2^iii^ = 3.8918 (15) Å and slippage = 1.551 Å; *Cg*2 is the centroid of the C7–C12 phenyl ring] contribute to the stabilization of the mol­ecular packing (Figs. 4 ▸ and 5 ▸).

## Hirshfeld surface analysis

**Figure 6:**
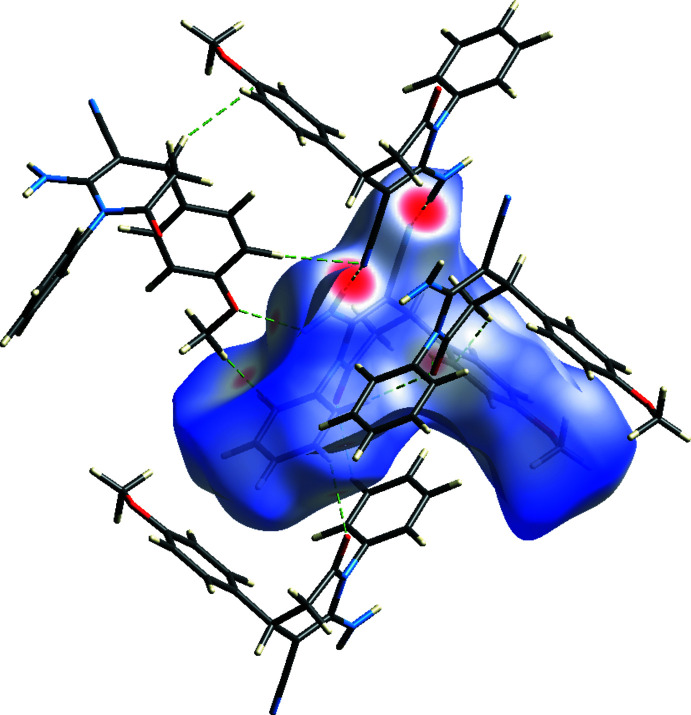
Hirshfeld surface mapped over *d*_norm_ showing the N—H⋯N, N—H⋯O and C—H⋯O inter­molecular contacts.

The Hirshfeld surface analysis was performed and the associated two dimensional fingerprint plots generated using *Crystal Explorer 17* (Turner *et al.*, 2017 ▸). The Hirshfeld surface was calculated using a standard (high) surface resolution with the three-dimensional *d*_norm_ surface plotted over a fixed colour scale mapped over the range −0.4835 (red) to 1.8469 (blue) a.u. The *d*_norm_ mapping indicates that strong hydrogen-bonding inter­actions, such as N—H⋯N, N—H⋯O and C—H⋯O hydrogen bonds (Tables 1 ▸ and 2 ▸), appear to be the primary inter­actions in the structure, seen as a bright-red area in the Hirshfeld surface (Fig. 6 ▸).

**Figure 7:**
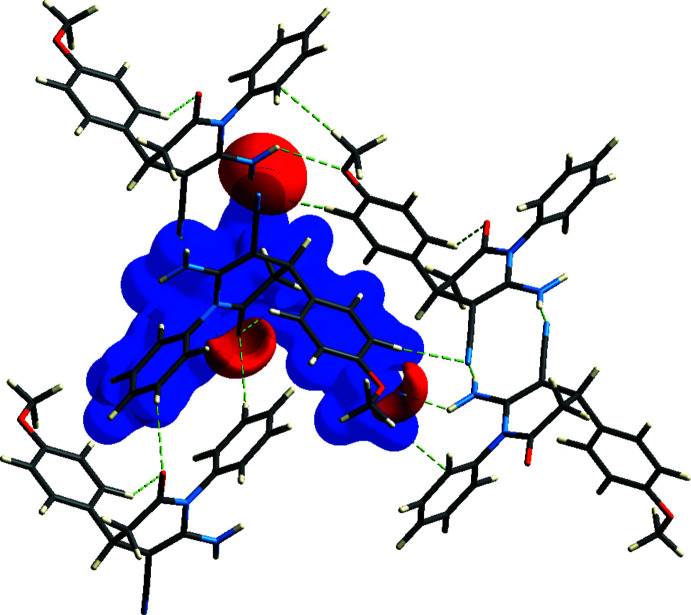
View of the three-dimensional Hirshfeld surface of the title compound, showing the hydrogen-bonding inter­actions, plotted over electrostatic potential energy in the range −0.0500 to 0.0500 a.u. using the STO-3 G basis set at the Hartree–Fock level of theory. Hydrogen-bond donors and acceptors are shown as blue and red regions, respectively, around the atoms, corresponding to positive and negative potentials.

The Hirshfeld surface mapped over electrostatic potential (Spackman *et al.*, 2008 ▸) is shown in Fig. 7 ▸. The blue regions indicate positive electrostatic potential (hydrogen-bond donors), while the red regions indicate negative electrostatic potential (hydrogen-bond acceptors).

**Figure 8:**
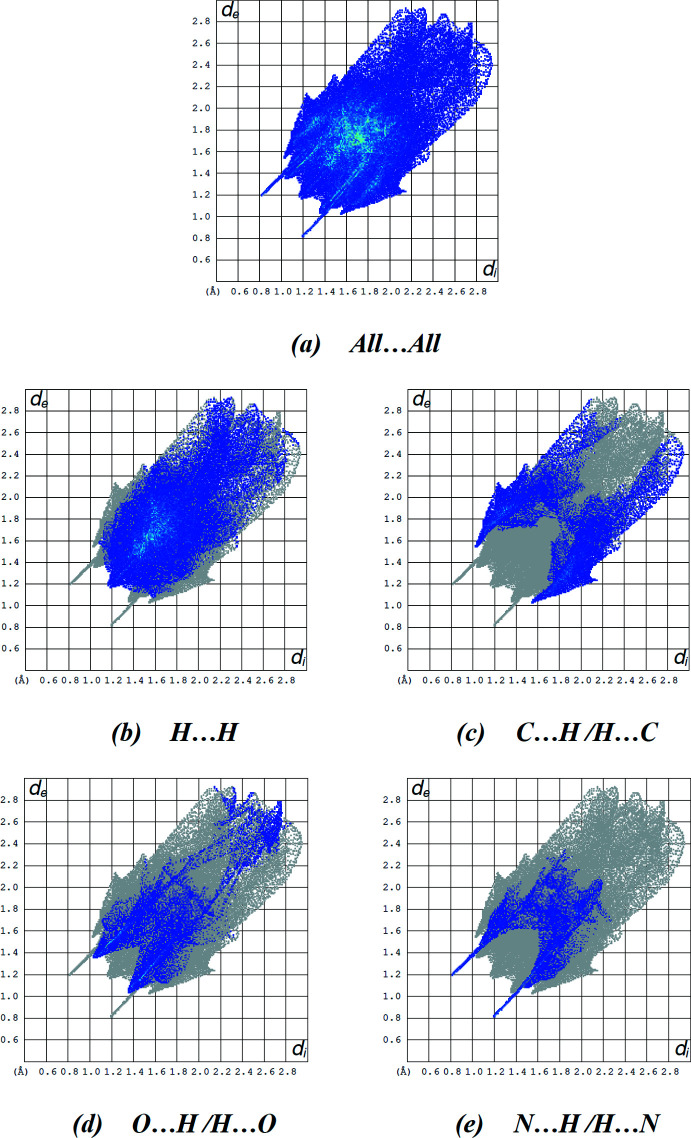
(*a*) The full two-dimensional fingerprint plot for the title compound and those delineated into (*b*) H⋯H (47.1%), (*c*) C⋯H/H⋯C (20.9%), (*d*) O⋯H/H⋯O (15.3%) and (*e*) N⋯H/H⋯N (11.4%) contacts.

The two-dimensional fingerprint plots are illustrated in Fig. 8 ▸. H⋯H contacts comprise 47.1% of the total inter­actions (Fig. 8 ▸ *b*), followed by C⋯H/H⋯C (Fig. 8 ▸ *c*; 20.9%), O⋯H/H⋯O (Fig. 8 ▸ *d*; 15.3%) and N⋯H/H⋯N (Fig. 8 ▸ *e*; 11.4%). The percentage contributions of the C⋯C, C⋯N/N⋯C and N⋯N contacts are negligible, at 3.1, 1.4 and 0.8%, respectively. The predominance of H⋯H, C⋯H/H⋯C, O⋯H/H⋯O and N⋯H/H⋯N contacts indicate that van der Waals inter­actions and hydrogen bonding play the major roles in the crystal packing (Hathwar *et al.*, 2015 ▸).

## Database survey

A search of the Cambridge Structural Database (CSD, version 5.42, update of September 2021; Groom *et al.*, 2016 ▸) found four compounds with the 6-oxo-1-phenyl-1,4,5,6-tetra­hydro­pyridine unit that are similar to the title compound, *viz*. 5-acetyl-2-amino-4-(4-bromo­phen­yl)-6-oxo-1-phenyl-1,4,5,6-tetra­hydro­pyridine-3-carbo­nitrile (**I**) (YAXQAT; Mamedov *et al.*, 2022 ▸), 2-amino-4-(2,6-di­chloro­phen­yl)-5-(1-hy­droxy­ethyl­idene)-6-oxo-1-phenyl-1,4,5,6-tetra­hydro­pyridine-3-car­b­o­nitrile (**II**) (OZAKOS, Naghiyev *et al.*, 2021*c* ▸), methyl 6-oxo-4-phenyl-2-[(*Z*)-2-(pyridin-2-yl)ethen­yl]-1,4,5,6-tetra­hydro­pyridine-3-carboxyl­ate (**III**) (PEDFEL, Smits *et al.*, 2012 ▸) and ethyl 5-eth­oxy­methyl­ene-2-methyl-6-oxo-4-phenyl-1,4,5,6-tetra­hydro­pyridine-3-carboxyl­ate (**IV**) (VAGXAD, Novoa de Armas *et al.*, 2003 ▸).

Compound (**I**) crystallizes in the monoclinic space group *P*c with *Z* = 4, and with two mol­ecules, *A* and *B*, in the asymmetric unit. These mol­ecules are stereoisomers with an *R*,*R* absolute configuration at C3 and C4 in mol­ecule *A*, whereas the corresponding atoms in *B*, C23 and C24, have an *S* configuration. In both mol­ecules, the conformation of the central di­hydro­pyridine ring is close to screw-boat. The mol­ecular conformation is stabilized by N—H⋯O hydrogen bonds, forming a dimer with an 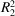 (16) ring motif. Both mol­ecules of the dimers are connected by inter­molecular N—H⋯O and N—H⋯N hydrogen bonds with an 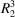 (14) ring motif into chains along the *c*-axis direction. Furthermore C—Br⋯π and C=O⋯π stacking inter­actions between these ribbons contribute to the stabilization of the mol­ecular packing.

Compound (**II**) crystallizes in the monoclinic space group *P*2_1_/*c* with *Z* = 4 and the asymmetric unit comprises one mol­ecule. The central tetra­hydro­pyridine ring is almost planar with a maximum deviation of 0.074 (3) Å for C4. The phenyl and di­chloro­phenyl rings are at an angle of 21.28 (15)°. They form dihedral angles of 86.10 (15) and 87.17 (14)°, respectively, with the central tetra­hydro­pyridine ring. A strong intra­molecular O2—H2⋯O1 hydrogen bond stabilizes the mol­ecular conformation of the mol­ecule, creating an *S*(6) ring motif. In the crystal, mol­ecules are linked by inter­molecular N—H⋯N and C—H⋯N hydrogen bonds, and N—H⋯π and C—H⋯π inter­actions, forming a three-dimensional network.

In mol­ecule (**III**) (monoclinic space group *P*2_1_/*c*, *Z* = 4), the *cis* configuration of the pyridinyl-vinyl fragment is stabilized by a strong intra­molecular N—H⋯N hydrogen bond. The phenyl and pyridine rings are inclined to one another by 77.3 (1)°. In the crystal, inversion dimers are present *via* pairs of C—H⋯O hydrogen bonds and are further linked by C—H⋯O hydrogen bonds and C—H⋯π inter­actions.

For compound (**IV**) (monoclinic space group *C*2/*c*, *Z* = 8), the mol­ecules form dimers by means of a pair of N—H⋯O hydrogen bonds. The 2(1*H*)-pyridone ring displays a screw-boat conformation.

## Synthesis and crystallization

To a solution of 2-(4-meth­oxy­benzyl­idene)malono­nitrile (0.94 g; 5.1 mmol) and acetoacetanilide (0.92 g; 5.2 mmol) in methanol (25 mL), 3-4 drops of piperidine were added and the mixture was stirred at 328–333 K for 10 min and was kept at room temperature for 48 h. Then 15 mL of methanol were removed from the reaction mixture, which was left overnight. The precipitated crystals were separated by filtration and recrystallized from ethanol/water (1:1) solution (yield 61%; m.p. 471–472 K).

^1^H NMR (300 MHz, DMSO-*d*_6_, ppm.): 2.80 (*dd*–*dd*, 1H, CH_2_); 3.19 (*dd*–*dd*, 1H, CH_2_); 3.82 (*s*, 3H, OCH_3_); 3.93 (*t*, 1H, CH); 5.85 (*s*, 2H, NH_2_); 7.15–7.58 (*m*, 9H, 2Ar—H). ^13^C NMR (75 MHz, DMSO-*d*_6_, ppm.): 36.06 (CH—Ar), 40.42 (CH_2_), 53.78 (OCH_3_), 59.05 (C_quat._), 112.89 (2CH_ar_), 121.21 (CN), 128.61 (CH_ar._), 128.88 (2CH_ar._), 130.44 (2CH_ar._), 130.51 (2CH_ar._), 136.06 (C_ar. quat._), 137.02 (C_ar. quat._), 154.59 (C_ar. quat._), 155.18 (C_quat._), 168.82 (N—C=O).

## Refinement details

Crystal data, data collection and structure refinement details are summarized in Table 3 ▸. H atoms bonded to nitro­gen were located in a difference-Fourier map, and only their positional parameters were refined [N3—H3*C* = 0.91 (2) and N3—H3*D* = 0.91 (2) Å with *U*_iso_(H) = 1.2*U*_eq_(N)]. C-bound H atoms were positioned geometrically, with C—H = 0.95–1.00 Å, and were refined with *U*_iso_(H) = 1.2*U*_eq_(C) or 1.5*U*_eq_(C-meth­yl).

## Supplementary Material

Crystal structure: contains datablock(s) I. DOI: 10.1107/S205698902200175X/vm2260sup1.cif

Structure factors: contains datablock(s) I. DOI: 10.1107/S205698902200175X/vm2260Isup2.hkl

Supporting information file. DOI: 10.1107/S205698902200175X/vm2260Isup3.cml

CCDC reference: 2152191

Additional supporting information:  crystallographic information; 3D view; checkCIF report

## Figures and Tables

**Figure 5:**
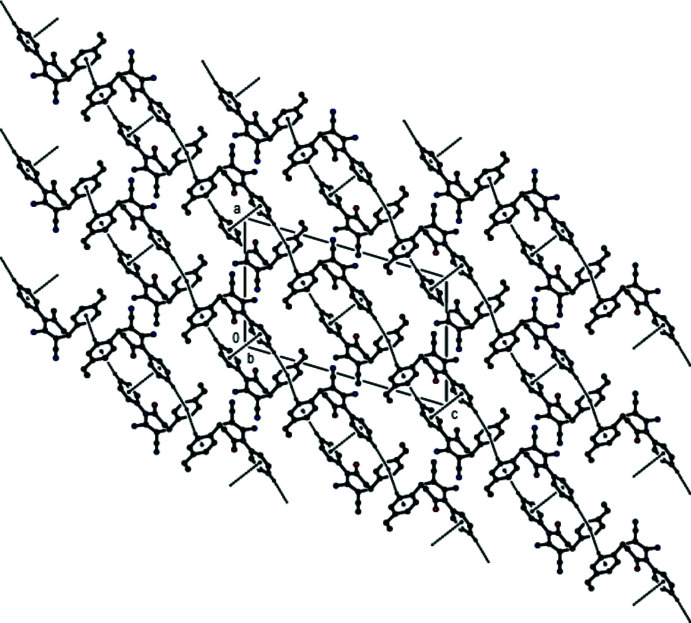
The crystal packing of the title compound, viewed along the *b* axis, showing the C—H⋯π inter­actions and π–π stacking inter­actions as dashed lines.

**Table 1:** Hydrogen-bond geometry (Å, °) *Cg*3 is the centroid of the C13–C18 benzene ring of the meth­oxy­phenyl group.

*D*—H⋯*A*	*D*—H	H⋯*A*	*D*⋯*A*	*D*—H⋯*A*
N3—H3*C*⋯N2^i^	0.91 (2)	2.10 (2)	2.996 (2)	166 (2)
N3—H3*D*⋯O2^ii^	0.91 (2)	2.48 (2)	3.152 (2)	131.0 (19)
C11—H11⋯O1^iii^	0.95	2.55	3.210 (3)	127
C14—H14⋯O1^iv^	0.95	2.48	3.199 (2)	133
C10—H10⋯*Cg*3^iii^	0.95	2.99	3.813 (3)	146
C18—H18⋯*Cg*3^v^	0.95	2.87	3.716 (2)	150

Symmetry codes: (i) 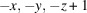 ; (ii) 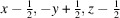 ; (iii) 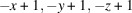 ; (iv) 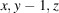 ; (v) 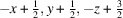 .

**Table 2:** Summary of short inter­atomic contacts (Å) in the title compound

Contact	Distance	Symmetry operation
O1⋯H14	2.48	*x*, 1 + *y*, *z*
H11⋯O1	2.55	1 − *x*, 1 − *y*, 1 − *z*
N2⋯H17	2.70	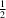 − *x*, − 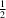 + *y*, 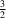 − *z*
O2⋯H3*D*	2.48 (2)	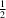 + *x*, 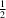 − *y*, 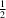 + *z*
H3*C*⋯N2	2.10 (3)	-*x*, −*y*, 1 − *z*
C20⋯C6	3.318 (3)	-*x*, 1 − *y*, 1 − *z*

**Table 3:** Experimental details

Crystal data	
Chemical formula	C_19_H_17_N_3_O_2_
*M*_r_	319.36
Crystal system, space group	Monoclinic, *P*2_1_/*n*
Temperature (K)	100
*a*, *b*, *c* (Å)	12.910 (3), 6.3200 (13), 21.170 (4)
β (°)	106.48 (3)
*V* (Å^3^)	1656.3 (7)
*Z*	4
Radiation type	Synchrotron, λ = 0.80246 Å
μ (mm^−1^)	0.11
Crystal size (mm)	0.40 × 0.15 × 0.07

Data collection	
Diffractometer	Rayonix SX165 CCD
Absorption correction	Multi-scan (*SCALA*;Evans, 2006 ▸)
*T*_min_, *T*_max_	0.950, 0.985
No. of measured, independent and observed [*I* > 2σ(*I*)] reflections	26143, 3603, 3125
*R*_int_	0.049
(sin θ/λ)_max_ (Å^−1^)	0.643

Refinement	
*R*[*F* ^2^ > 2σ(*F* ^2^)], *wR*(*F* ^2^), *S*	0.054, 0.143, 1.05
No. of reflections	3603
No. of parameters	225
H-atom treatment	H atoms treated by a mixture of independent and constrained refinement
Δρ_max_, Δρ_min_ (e Å^−3^)	0.29, −0.27

Computer programs: *Marccd* (Doyle, 2011 ▸), *iMosflm* (Battye *et al.*, 2011 ▸), *SHELXT* (Sheldrick, 2015*a* ▸), *SHELXL* (Sheldrick, 2015*b* ▸), *ORTEP-3 for Windows* (Farrugia, 2012 ▸) and *PLATON* (Spek, 2020 ▸).

## supplementary crystallographic information

### Crystal data

**Table:**

C_19_H_17_N_3_O_2_	*F*(000) = 672
*M**_r_* = 319.36	*D*_x_ = 1.281 Mg m^−^^3^
Monoclinic, *P*2_1_/*n*	Synchrotron radiation, λ = 0.80246 Å
*a* = 12.910 (3) Å	Cell parameters from 600 reflections
*b* = 6.3200 (13) Å	θ = 2.4–30.0°
*c* = 21.170 (4) Å	µ = 0.11 mm^−^^1^
β = 106.48 (3)°	*T* = 100 K
*V* = 1656.3 (7) Å^3^	Prism, colourless
*Z* = 4	0.40 × 0.15 × 0.07 mm

### Data collection

**Table:**

Rayonix SX165 CCD diffractometer	3125 reflections with *I* > 2σ(*I*)
/f scan	*R*_int_ = 0.049
Absorption correction: multi-scan (Scala;Evans, 2006)	θ_max_ = 31.1°, θ_min_ = 2.3°
*T*_min_ = 0.950, *T*_max_ = 0.985	*h* = −16→16
26143 measured reflections	*k* = −8→7
3603 independent reflections	*l* = −27→27

### Refinement

**Table:**

Refinement on *F*^2^	Secondary atom site location: difference Fourier map
Least-squares matrix: full	Hydrogen site location: mixed
*R*[*F*^2^ > 2σ(*F*^2^)] = 0.054	H atoms treated by a mixture of independent and constrained refinement
*wR*(*F*^2^) = 0.143	*w* = 1/[σ^2^(*F*_o_^2^) + (0.059*P*)^2^ + 1.3785*P*] where *P* = (*F*_o_^2^ + 2*F*_c_^2^)/3
*S* = 1.05	(Δ/σ)_max_ < 0.001
3603 reflections	Δρ_max_ = 0.29 e Å^−^^3^
225 parameters	Δρ_min_ = −0.27 e Å^−^^3^
0 restraints	Extinction correction: SHELXL, Fc^*^=kFc[1+0.001xFc^2^λ^3^/sin(2θ)]^-1/4^
Primary atom site location: difference Fourier map	Extinction coefficient: 0.033 (3)

### Special details

**Table:**

Geometry. All esds (except the esd in the dihedral angle between two l.s. planes) are estimated using the full covariance matrix. The cell esds are taken into account individually in the estimation of esds in distances, angles and torsion angles; correlations between esds in cell parameters are only used when they are defined by crystal symmetry. An approximate (isotropic) treatment of cell esds is used for estimating esds involving l.s. planes.

### Fractional atomic coordinates and isotropic or equivalent isotropic displacement parameters (Å^2^)

**Table:**

	*x*	*y*	*z*	*U*_iso_*/*U*_eq_	
O1	0.33220 (11)	0.8608 (2)	0.55256 (7)	0.0332 (3)	
O2	0.55206 (12)	0.1472 (3)	0.79862 (7)	0.0413 (4)	
N1	0.24126 (11)	0.5628 (2)	0.50951 (7)	0.0269 (3)	
N2	−0.03015 (13)	0.1368 (3)	0.56257 (8)	0.0321 (4)	
N3	0.12078 (12)	0.3027 (3)	0.45307 (8)	0.0300 (4)	
H3C	0.0857 (18)	0.177 (4)	0.4520 (11)	0.036*	
H3D	0.1458 (19)	0.336 (4)	0.4183 (12)	0.036*	
C2	0.26361 (14)	0.7307 (3)	0.55371 (9)	0.0272 (4)	
C3	0.19790 (14)	0.7407 (3)	0.60221 (9)	0.0287 (4)	
H3A	0.1284	0.8122	0.5814	0.034*	
H3B	0.2372	0.8254	0.6409	0.034*	
C4	0.17589 (14)	0.5196 (3)	0.62495 (9)	0.0277 (4)	
H4	0.1221	0.5339	0.6506	0.033*	
C5	0.12391 (13)	0.3942 (3)	0.56355 (9)	0.0268 (4)	
C6	0.15916 (13)	0.4149 (3)	0.50888 (9)	0.0262 (4)	
C7	0.30690 (13)	0.5355 (3)	0.46514 (9)	0.0279 (4)	
C8	0.31570 (15)	0.6969 (3)	0.42287 (9)	0.0338 (4)	
H8	0.2765	0.8249	0.4215	0.041*	
C9	0.38256 (17)	0.6690 (4)	0.38261 (11)	0.0408 (5)	
H9	0.3906	0.7800	0.3541	0.049*	
C10	0.43779 (17)	0.4806 (4)	0.38357 (11)	0.0421 (5)	
H10	0.4827	0.4624	0.3554	0.051*	
C11	0.42768 (16)	0.3194 (4)	0.42533 (10)	0.0379 (5)	
H11	0.4651	0.1898	0.4256	0.045*	
C12	0.36267 (15)	0.3466 (3)	0.46698 (9)	0.0314 (4)	
H12	0.3564	0.2370	0.4964	0.038*	
C13	0.27801 (14)	0.4188 (3)	0.66992 (9)	0.0266 (4)	
C14	0.32417 (14)	0.2403 (3)	0.65102 (9)	0.0274 (4)	
H14	0.2927	0.1822	0.6085	0.033*	
C15	0.41514 (15)	0.1438 (3)	0.69241 (9)	0.0298 (4)	
H15	0.4443	0.0197	0.6787	0.036*	
C16	0.46259 (15)	0.2306 (3)	0.75379 (9)	0.0316 (4)	
C17	0.41891 (15)	0.4125 (3)	0.77340 (9)	0.0333 (4)	
H17	0.4520	0.4735	0.8153	0.040*	
C18	0.32761 (15)	0.5043 (3)	0.73205 (9)	0.0301 (4)	
H18	0.2980	0.6273	0.7460	0.036*	
C19	0.5911 (2)	−0.0507 (4)	0.78134 (12)	0.0535 (6)	
H19A	0.6153	−0.0321	0.7418	0.080*	
H19B	0.5329	−0.1558	0.7726	0.080*	
H19C	0.6517	−0.0995	0.8178	0.080*	
C20	0.03871 (13)	0.2533 (3)	0.56225 (9)	0.0268 (4)	

### Atomic displacement parameters (Å^2^)

**Table:**

	*U*^11^	*U*^22^	*U*^33^	*U*^12^	*U*^13^	*U*^23^
O1	0.0328 (7)	0.0278 (7)	0.0408 (7)	−0.0063 (5)	0.0132 (6)	−0.0045 (6)
O2	0.0378 (7)	0.0485 (9)	0.0319 (7)	0.0068 (6)	0.0005 (6)	0.0031 (6)
N1	0.0262 (7)	0.0245 (7)	0.0304 (7)	−0.0025 (6)	0.0086 (6)	−0.0026 (6)
N2	0.0318 (8)	0.0294 (8)	0.0348 (8)	−0.0028 (6)	0.0093 (6)	0.0009 (7)
N3	0.0311 (8)	0.0297 (8)	0.0288 (8)	−0.0072 (6)	0.0079 (6)	−0.0025 (6)
C2	0.0272 (8)	0.0218 (8)	0.0310 (9)	0.0010 (6)	0.0058 (7)	0.0001 (7)
C3	0.0300 (8)	0.0248 (8)	0.0319 (9)	0.0000 (7)	0.0096 (7)	−0.0022 (7)
C4	0.0267 (8)	0.0268 (9)	0.0305 (9)	−0.0005 (7)	0.0098 (7)	−0.0023 (7)
C5	0.0240 (8)	0.0255 (8)	0.0300 (9)	−0.0003 (6)	0.0060 (6)	0.0015 (7)
C6	0.0233 (7)	0.0228 (8)	0.0303 (8)	−0.0006 (6)	0.0040 (6)	0.0003 (7)
C7	0.0243 (8)	0.0310 (9)	0.0278 (8)	−0.0053 (7)	0.0064 (6)	−0.0040 (7)
C8	0.0319 (9)	0.0351 (10)	0.0331 (9)	−0.0054 (8)	0.0072 (7)	0.0005 (8)
C9	0.0395 (10)	0.0479 (12)	0.0366 (10)	−0.0109 (9)	0.0135 (8)	0.0021 (9)
C10	0.0368 (10)	0.0531 (13)	0.0404 (11)	−0.0106 (9)	0.0173 (9)	−0.0088 (10)
C11	0.0304 (9)	0.0418 (11)	0.0426 (11)	−0.0044 (8)	0.0122 (8)	−0.0118 (9)
C12	0.0296 (9)	0.0313 (9)	0.0329 (9)	−0.0019 (7)	0.0083 (7)	−0.0033 (8)
C13	0.0279 (8)	0.0239 (8)	0.0286 (8)	−0.0032 (6)	0.0089 (7)	−0.0008 (7)
C14	0.0276 (8)	0.0259 (8)	0.0278 (8)	−0.0031 (7)	0.0067 (7)	−0.0011 (7)
C15	0.0308 (8)	0.0283 (9)	0.0312 (9)	0.0005 (7)	0.0105 (7)	0.0018 (7)
C16	0.0300 (9)	0.0356 (10)	0.0273 (9)	−0.0003 (7)	0.0047 (7)	0.0052 (7)
C17	0.0347 (9)	0.0368 (10)	0.0269 (9)	−0.0047 (8)	0.0063 (7)	−0.0033 (8)
C18	0.0342 (9)	0.0280 (9)	0.0290 (9)	−0.0020 (7)	0.0106 (7)	−0.0030 (7)
C19	0.0529 (13)	0.0586 (15)	0.0408 (12)	0.0229 (12)	0.0002 (10)	0.0033 (11)
C20	0.0264 (8)	0.0245 (8)	0.0280 (8)	0.0024 (7)	0.0054 (6)	0.0005 (7)

### Geometric parameters (Å, º)

**Table:**

O1—C2	1.214 (2)	C8—H8	0.9500
O2—C16	1.375 (2)	C9—C10	1.385 (3)
O2—C19	1.434 (3)	C9—H9	0.9500
N1—C2	1.390 (2)	C10—C11	1.379 (3)
N1—C6	1.410 (2)	C10—H10	0.9500
N1—C7	1.443 (2)	C11—C12	1.390 (3)
N2—C20	1.156 (2)	C11—H11	0.9500
N3—C6	1.346 (2)	C12—H12	0.9500
N3—H3C	0.91 (2)	C13—C14	1.387 (2)
N3—H3D	0.91 (2)	C13—C18	1.398 (3)
C2—C3	1.507 (2)	C14—C15	1.391 (3)
C3—C4	1.530 (2)	C14—H14	0.9500
C3—H3A	0.9900	C15—C16	1.383 (3)
C3—H3B	0.9900	C15—H15	0.9500
C4—C5	1.508 (2)	C16—C17	1.395 (3)
C4—C13	1.529 (2)	C17—C18	1.381 (3)
C4—H4	1.0000	C17—H17	0.9500
C5—C6	1.365 (2)	C18—H18	0.9500
C5—C20	1.410 (2)	C19—H19A	0.9800
C7—C8	1.383 (3)	C19—H19B	0.9800
C7—C12	1.389 (3)	C19—H19C	0.9800
C8—C9	1.386 (3)		
			
C16—O2—C19	116.44 (16)	C8—C9—H9	119.7
C2—N1—C6	121.62 (15)	C11—C10—C9	120.19 (19)
C2—N1—C7	118.78 (14)	C11—C10—H10	119.9
C6—N1—C7	119.55 (14)	C9—C10—H10	119.9
C6—N3—H3C	122.5 (15)	C10—C11—C12	119.9 (2)
C6—N3—H3D	117.7 (15)	C10—C11—H11	120.0
H3C—N3—H3D	118 (2)	C12—C11—H11	120.0
O1—C2—N1	121.14 (16)	C7—C12—C11	119.35 (19)
O1—C2—C3	122.71 (16)	C7—C12—H12	120.3
N1—C2—C3	116.16 (15)	C11—C12—H12	120.3
C2—C3—C4	111.53 (14)	C14—C13—C18	117.78 (17)
C2—C3—H3A	109.3	C14—C13—C4	121.64 (16)
C4—C3—H3A	109.3	C18—C13—C4	120.58 (16)
C2—C3—H3B	109.3	C13—C14—C15	122.01 (17)
C4—C3—H3B	109.3	C13—C14—H14	119.0
H3A—C3—H3B	108.0	C15—C14—H14	119.0
C5—C4—C13	114.33 (15)	C16—C15—C14	119.18 (17)
C5—C4—C3	106.59 (15)	C16—C15—H15	120.4
C13—C4—C3	111.84 (14)	C14—C15—H15	120.4
C5—C4—H4	108.0	O2—C16—C15	123.92 (18)
C13—C4—H4	108.0	O2—C16—C17	116.22 (17)
C3—C4—H4	108.0	C15—C16—C17	119.86 (17)
C6—C5—C20	119.33 (16)	C18—C17—C16	120.13 (17)
C6—C5—C4	120.46 (15)	C18—C17—H17	119.9
C20—C5—C4	120.21 (16)	C16—C17—H17	119.9
N3—C6—C5	124.45 (16)	C17—C18—C13	121.01 (18)
N3—C6—N1	116.48 (16)	C17—C18—H18	119.5
C5—C6—N1	119.07 (16)	C13—C18—H18	119.5
C8—C7—C12	121.01 (17)	O2—C19—H19A	109.5
C8—C7—N1	120.29 (17)	O2—C19—H19B	109.5
C12—C7—N1	118.68 (16)	H19A—C19—H19B	109.5
C7—C8—C9	118.95 (19)	O2—C19—H19C	109.5
C7—C8—H8	120.5	H19A—C19—H19C	109.5
C9—C8—H8	120.5	H19B—C19—H19C	109.5
C10—C9—C8	120.5 (2)	N2—C20—C5	178.6 (2)
C10—C9—H9	119.7		
			
C6—N1—C2—O1	−177.75 (16)	C12—C7—C8—C9	−0.9 (3)
C7—N1—C2—O1	4.8 (3)	N1—C7—C8—C9	177.59 (17)
C6—N1—C2—C3	2.1 (2)	C7—C8—C9—C10	1.5 (3)
C7—N1—C2—C3	−175.40 (15)	C8—C9—C10—C11	−0.8 (3)
O1—C2—C3—C4	−143.36 (18)	C9—C10—C11—C12	−0.6 (3)
N1—C2—C3—C4	36.8 (2)	C8—C7—C12—C11	−0.4 (3)
C2—C3—C4—C5	−54.71 (18)	N1—C7—C12—C11	−178.91 (16)
C2—C3—C4—C13	70.92 (19)	C10—C11—C12—C7	1.1 (3)
C13—C4—C5—C6	−84.4 (2)	C5—C4—C13—C14	7.1 (2)
C3—C4—C5—C6	39.7 (2)	C3—C4—C13—C14	−114.15 (18)
C13—C4—C5—C20	95.23 (19)	C5—C4—C13—C18	−172.53 (16)
C3—C4—C5—C20	−140.67 (16)	C3—C4—C13—C18	66.2 (2)
C20—C5—C6—N3	−2.7 (3)	C18—C13—C14—C15	1.7 (3)
C4—C5—C6—N3	176.95 (17)	C4—C13—C14—C15	−177.99 (16)
C20—C5—C6—N1	177.30 (15)	C13—C14—C15—C16	−1.4 (3)
C4—C5—C6—N1	−3.1 (2)	C19—O2—C16—C15	−5.2 (3)
C2—N1—C6—N3	159.46 (16)	C19—O2—C16—C17	174.22 (19)
C7—N1—C6—N3	−23.1 (2)	C14—C15—C16—O2	179.53 (17)
C2—N1—C6—C5	−20.5 (2)	C14—C15—C16—C17	0.1 (3)
C7—N1—C6—C5	156.97 (16)	O2—C16—C17—C18	−178.57 (17)
C2—N1—C7—C8	−57.2 (2)	C15—C16—C17—C18	0.9 (3)
C6—N1—C7—C8	125.25 (18)	C16—C17—C18—C13	−0.6 (3)
C2—N1—C7—C12	121.29 (18)	C14—C13—C18—C17	−0.6 (3)
C6—N1—C7—C12	−56.3 (2)	C4—C13—C18—C17	179.03 (17)

### Hydrogen-bond geometry (Å, º)

*Cg*3 is the centroid of the C13–C18 benzene ring of the methoxyphenyl group.

**Table:**

*D*—H···*A*	*D*—H	H···*A*	*D*···*A*	*D*—H···*A*
N3—H3*C*···N2^i^	0.91 (2)	2.10 (2)	2.996 (2)	166 (2)
N3—H3*D*···O2^ii^	0.91 (2)	2.48 (2)	3.152 (2)	131.0 (19)
C11—H11···O1^iii^	0.95	2.55	3.210 (3)	127
C14—H14···O1^iv^	0.95	2.48	3.199 (2)	133
C10—H10···*Cg*3^iii^	0.95	2.99	3.813 (3)	146
C18—H18···*Cg*3^v^	0.95	2.87	3.716 (2)	150

Symmetry codes: (i) −*x*, −*y*, −*z*+1; (ii) *x*−1/2, −*y*+1/2, *z*−1/2; (iii) −*x*+1, −*y*+1, −*z*+1; (iv) *x*, *y*−1, *z*; (v) −*x*+1/2, *y*+1/2, −*z*+3/2.
